# Optimized GA_3_ Production by *Aspergillus niger* and *Fusarium fujikuroi*: From Low-Cost Fermentation to Agronomic Application

**DOI:** 10.1021/acsomega.5c09459

**Published:** 2025-12-13

**Authors:** Fabrício Gomes Menezes Porto, Welisson Martins Rocha, Adrielle Aparecida Paulista Ribeiro, Amanda Carmelo da Rocha, Iara Rossi Gonçalves, Eloízio Júlio Ribeiro, Miriam Maria de Resende

**Affiliations:** † School of Chemical Engineering, Federal University of Uberlândia, Campus Santa Mônica, Uberlândia-MG 38400-902, Brazil; ‡ Satis Indústria e Comércio LTDA, Araxá-MG 38183-190, Brazil; § Department of Chemical Engineering, University of Uberaba, Uberaba-MG 38055-500, Brazil

## Abstract

This study aimed to optimize low-cost submerged fermentation for gibberellic acid (GA_3_) production using *Aspergillus niger* LGB-034–2015 and *Fusarium fujikuroi* Insumicro 175, followed by evaluating the fermentation supernatant in common bean (*Phaseolus vulgaris*) under field conditions. A factorial design and central composite design were applied to test different concentrations of glucose (10–30 g/L), glycerol (5–15 g/L), and yeast extract (2–6 g/L). Maximum GA_3_ production reached 597.03 mg/L (0.60 g/L) for *A. niger* and 487.23 mg/L (0.49 g/L) for *F. fujikuroi*. The highest yield per unit of substrate consumed was 550 mg of GA_3_ per gram of glucose by *A. niger*. In the agronomic trials, the supernatant was applied as a foliar spray at 200 mL per plot (four applications during the crop cycle) and compared with untreated controls. The results showed a significant reduction in activity of antioxidant enzymes, such as superoxide dismutase (SOD), catalase (CAT), and peroxidase (POD), and an increase in yield, reaching 44.8 bags per hectare. These findings demonstrate that optimizing GA_3_ fermentation with *A. niger* is a cost-effective approach with practical applications in crop productivity.

## Graphical abstract

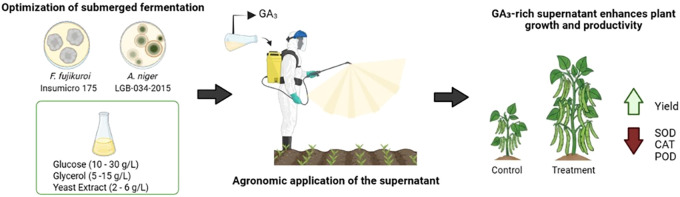

## 1 Introduction

Gibberellic acid (GA_3_) is a vital plant growth regulator with recognized roles in promoting stem elongation, seed germination, and fruit development and is widely applied in agriculture to improve crop performance.^1,2^ GA_3_ is a key organic chemical used by commercial growers due to its rapid impact on both vegetative and floral yields, demonstrating its value in enhancing product quality and marketability. The global demand for effective and cost-efficient biostimulants positions GA_3_ as a high-value agricultural input.^3^ Traditionally, GA_3_ has been produced through submerged fermentation using fungal strains of *Fusarium fujikuroi*, a filamentous fungus initially discovered in the 1920s (as *Gibberella fujikoroi*) and known for its ability to biosynthesize high levels of this phytohormone. For many years, this species was considered the only fungal source of GA_3_; however, subsequent studies have demonstrated that other *Fusarium* species also possess this biosynthetic capability.^4,5^ Moreover, other fungi, such as *Aspergillus* spp. and *Penicillium* spp., as well as several bacteria, including *Azospirillum* spp., *Pseudomonas* spp., and *Rhizobium* spp., are also capable of synthesizing this phytohormone under specific conditions.^6^

Despite the advances in microbial GA_3_ production, challenges remain related to process yield, substrate cost, and scalability.^7–9^ The exploration of alternative microorganisms, nonenriched media, and optimized culture conditions is essential to reduce production costs and expand the biotechnological use of GA_3_, especially in the development of sustainable agricultural inputs. In this context, strains of *Aspergillus niger*, a well-studied organism, have shown potential for GA_3_ biosynthesis, yet their performance under optimized, low-cost conditions remains underexplored.^10,11^

Moreover, GA_3_-containing fermentation supernatants may offer additional advantages beyond phytohormone production, functioning as natural biostimulants capable of improving crop resilience to abiotic stress.^12,13^ Recent studies suggest that such bioproducts can reduce oxidative damage and increase yield in several crops, positioning them as sustainable alternatives to synthetic inputs.^1,14,15^

Therefore, this study aimed to evaluate and optimize GA_3_ production by *A. niger* and *F. fujikuroi* using simple substrates under submerged fermentation. Additionally, we investigated the agronomic effects of the fermentation supernatants on common beans (*Phaseolus vulgaris*), assessing their impact on oxidative stress markers, enzymatic activity, and productivity under field conditions. The results contribute to expanding the range of microbial producers of GA_3_ and reinforce the dual potential of microbial supernatants as both bioproducts and biostimulants in agriculture.

## 2 Materials and Methods

### 2.1 Fungal Strains and Culture

The fungi *Fusarium fujikuroi* (Insumicro 175 strain) and *Aspergillus niger* (LGB-034–2015 strain), provided by Embrapa Agroenergia, were evaluated in this study as potential producers of gibberellic acid (GA_3_). The strains were preserved following Castellani’s method.^16^ Microorganisms’ reactivation was performed by subculturing the cultures in Petri dishes containing the BDA (Potato-Dextrose-Agar) medium. The plates were incubated at 28 °C for 7 days, with a 12 h photoperiod.

### 2.2 Submerged Fermentation in a Shaker Incubator

To optimize the bioprocess for an enhanced GA_3_ concentration, a comprehensive Central Composite Design (CCD) was implemented. The concentrations of glucose, glycerol, and yeast extract were designated as independent variables, with their axial ranges defined based on literature-established values for GA_3_ production.^17–19^ This approach, using well-defined substrates, allowed for a comprehensive and precise evaluation of their singular and synergistic effects on the fermentation process, which is critical for maximizing conversion efficiency and cost-effectiveness.

The CCD, encompassing the evaluated factor levels and central points, is presented in Table 1. The experimental trials were conducted for both fungal strains under investigation: Insumicro 175 and LGB-034–2015. A total of 68 distinct experimental conditions were duplicated and performed, including three central points. The samples were collected at designated time points of 72, 96, 120, and 144 h, with the latter representing the final sampling point. After fermentation, the concentrations of GA_3_, residual glucose, and fungal dry matter were quantified. For statistical analyses, the GA_3_ production data obtained at 144 h were considered, as this time point consistently yielded the highest metabolite concentrations. The yield of product per substrate consumed (*Y*_P/S_) was calculated using eq 1.

1 $$Y_{p/s}=\frac{\left[{\mathrm{GA}}_{3}\right]\left(\mathrm{mg}/L\right)}{\left[g\mathrm{lucose}\right]\left(g/L\right)}$$

**Table 1:** Central Composite Design Applied to *Aspergillus niger* (LGB-034-201) and *Fusarium fujikuroi* (Insumicro 175) Fermentations^a^

Trial	Glucose (g/L)	Glycerol (g/L)	Yeast extract (g/L)
1	40.0	2.0	2.0
2	40.0	2.0	7.0
3	40.0	7.0	2.0
4	40.0	7.0	7.0
5	80.0	2.0	2.0
6	80.0	2.0	7.0
7	80.0	7.0	2.0
8	80.0	7.0	7.0
9	26.4	4.5	4.5
10	93.6	4.5	4.5
11	60.0	0.0	4.5
12	60.0	8.7	4.5
13	60.0	4.5	0.0
14	60.0	4.5	8.7
15 (CP)	60.0	4.5	4.5
16 (CP)	60.0	4.5	4.5
17 (CP)	60.0	4.5	4.5

a CP, central point.

### 2.3 Evaluation of Supernatant Influence on Plant Stress Physiology and Bean Yield

The experiment was conducted at the Satis Experimental Field, situated in the municipality of Araxá, Alto Paranaíba Region, Minas Gerais. This location is precisely at 19°33′5.20′S latitude and 46°52′33.65′W longitude, with an altitude of 900 m. According to Koeppenʼs classification, the regionʼs climate is characterized as Cwa, indicating a temperate humid climate with dry winters and hot summers. Figure 1 illustrates the monthly accumulated rainfall regime during the 2023–2024 harvest period, with data acquired from the meteorological station installed at the Satis Experimental Field.

**Figure 1:**
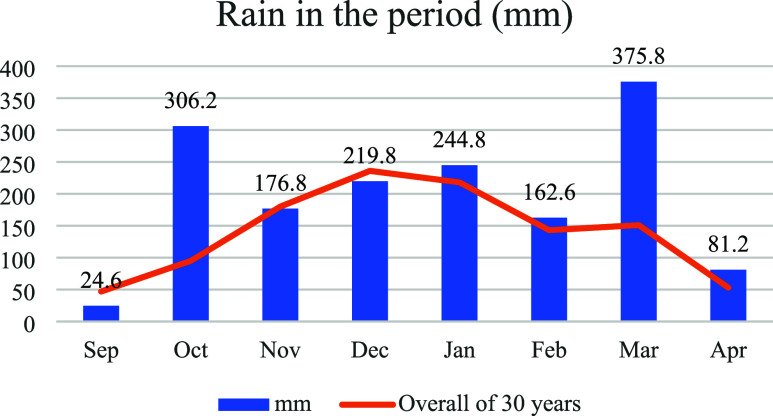
Monthly accumulated rainfall during the 2023–2024 harvest period at the experimental site.

The experimental site was classified as silt clay Latossol. Baseline soil fertility before the experiment showed organic matter content of 34.1 g/dm^3^ and available phosphorus (P) at 30.3 mg/dm^3^ and potassium (K) at 4.4 mmolc/dm^3^. The preceding crop was soybeans.

The assay was conducted using the carioca common bean IAC 1850 cultivar. Plots, each measuring 6 m in length by 2 m in width, were allocated within a randomized design. Planting occurred on January 10, 2024, and harvesting occurred on May 10, 2024. Treatments, detailed in Table 2, included a control (no GA_3_ application), a positive control (commercial GA_3_ product at 400 g/kg, applied according to manufacturer’s recommendations), and four different doses of the fermentation supernatant (SF) obtained from the CCD. Each treatment was replicated four times (*n* = 4), resulting in a total of 24 experimental units. Foliar applications were carried out using a backpack sprayer pressurized with CO_2_ at a constant pressure of 30 psi. Leaf samples were collected 3 days after the second application for comprehensive evaluations of the enzymatic activities of catalase (CAT), superoxide dismutase (SOD), and peroxidase (POD), all enzymes linked to plant stress physiology, as well as lipid peroxidation (LP) and total soluble protein (TSP). Upon completion of the crop cycle, the central rows of each plot were harvested to quantify productivity in bags per hectare and the thousand-grain weight. The resulting data underwent statistical analysis using the Scott–Knott test (*P* < 0.05), chosen for its ability to separate means into nonoverlapping groups, ensuring clearer interpretation of treatment effects.

**Table 2:** Experimental Design for Evaluating the Agronomic Effect of the Supernatant on the Bean Crop^a^

Treatments	Doses (mL or g/ha)	Application time
Control		
Positive Control	5.00	R5 + 7–10 days
SF	250	R5 + 7–10 days
SF	500	R5 + 7–10 days
SF	1.00 × 10^3^	R5 + 7–10 days
SF	2.00 × 10^3^	R5 + 7–10 days

a SF, fermentation supernatant.

### 2.4 Statistical Analysis, Graphs, and Flowcharts

Statistical analyses were performed using Statistica software, versions 7 and 13, from Statsoft. Graphs were plotted using OriginPro 2018 software from OriginLab Corporation, and flowcharts were drawn using the Biorender application.

After sampling, the broth was filtered to remove the biomass. A 10 mL aliquot was clarified by precipitating macromolecules with 0.5 mL of 30% zinc acetate and 0.5 mL of 15% potassium ferrocyanide, followed by centrifugation. One milliliter of the supernatant was transferred to a 10 mL volumetric flask, 1 mL of ethanol was added, and the volume was completed with 30% HCl to convert gibberellic acid to gibberellenic acid; the solution was kept at rest for 75 min and read at 254 nm on a calibrated spectrophotometer.^20^ GA_3_ concentration was obtained from a calibration curve prepared with a 90% GA_3_ commercial standard (Neon) dissolved in ethanol at 1, 3, 6, 9, 15, and 18 ppm, yielding *R* ^2^ = 0.99. Method validation, in which a 100 ppm of GA_3_ standard was carried through the full procedure (including the zinc acetate and potassium ferrocyanide steps), showed 98% recovery, indicating negligible matrix interference.

## 3 Results

### 3.1 Central Composite Design (CCD): Influence of Combined Variables at Different Concentrations

The fungal strains were subjected to a CCD combining different concentrations of the main factors studied in this work. Table 3 presents the values obtained for GA_3_ production, residual glucose, and fermentation yield from *A. niger* strain LGB-034–2015.

**Table 3:** Central Composite Design Matrix Evaluating the Effects of Glucose, Glycerol, and Yeast Extract Concentrations on Gibberellic Acid (GA_3_) Production, Residual Glucose, and Product Yield (*Y*_P/S_) by *Aspergillus niger* Strain LGB-034-2015^a^

Trial	Glucose (g/L)	Glycerol (g/L)	Yeast extract (g/L)	GA_3_ (mg/L)	Residual glucose (g/L)	Y_P/S_ (mg/g)
1	40.0	2.0	2.0	42.690	21.80	2.560
2	40.0	2.0	7.0	382.61	14.59	15.06
3	40.0	7.0	2.0	75.670	27.88	3.650
4	40.0	7.0	7.0	394.23	21.22	44.63
5	80.0	2.0	2.0	63.910	63.37	3.160
6	80.0	2.0	7.0	376.67	58.12	58.56
7	80.0	7.0	2.0	92.060	69.55	8.810
8	80.0	7.0	7.0	377.98	62.17	14.90
9	26.4	4.5	4.5	169.16	4.340	8.030
10	93.6	4.5	4.5	234.08	57.16	6.420
11	60.0	0.0	4.5	198.78	40.36	10.12
12	60.0	8.7	4.5	250.27	51.98	31.21
13	60.0	4.5	0.0	41.870	48.61	2.550
14	60.0	4.5	8.7	546.89	39.11	32.39
15 (CP)	60.0	4.5	4.5	230.71	45.54	15.95
16 (CP)	60.0	4.5	4.5	239.75	46.68	18.00
17 (CP)	60.0	4.5	4.5	228.75	46.75	17.27

a GA_3_, gibberellic acid; CP, central point; *Y*_P/S_, product yield with glucose.

A substantial variation in GA_3_ production was observed, with concentrations ranging from 41.870 mg/L (trial 13) to 546.89 mg/L (trial 14). Residual glucose values varied between 4.340 g/L (trial 9) and 69.55 g/L (trial 7). The product yield with glucose (*Y*_P/S_) ranged from 2.550 mg/g (trial 13) to 58.56 mg/g (trial 6).

Among the trials with a low initial glucose concentration (40 g/L), GA_3_ production ranged from 42.690 to 394.23 mg/L. In trials with a high glucose concentration (80 g/L), values ranged from 63.910 to 377.98 mg/L. Intermediate glucose levels (60 g/L) were tested in combination with different glycerol and yeast extract concentrations, resulting in GA_3_ production values ranging from 41.87 to 546.89 mg/L.

Trials 9 and 10 tested axial levels of glucose (26.4 and 93.6 g/L, respectively) with fixed levels of the other two components. GA_3_ concentrations under these conditions were 169.16 and 234.08 mg/L, respectively. Trials 11 and 12 explored the lower and upper axial levels of glycerol (0.0 and 8.7 g/L), while trials 13 and 14 varied yeast extract from 0.0 to 8.7 g/L. The central point (trials 15 to 17), performed in triplicate to assess model reproducibility, resulted in GA_3_ concentrations of 228.75, 230.71, and 239.75 mg/L, with residual glucose ranging from 45.54 to 46.75 g/L and *Y*_P/S_ values between 15.95 and 18.00 mg/g.

The response surface plots (Figure 2) illustrate the interactions between the substrate components and their influence on GA_3_ production by the *A. niger* strain LGB-034–2015 strain. At higher concentrations of yeast extract, the effects of both glycerol and glucose on GA_3_ production become less pronounced. In all evaluated substrate conditions, the use or increase in the concentration of glycerol reduced the level of GA_3_ production. However, when glycerol was used at low concentrations in combination with yeast extract, it contributed to higher levels of GA_3_ production. Additionally, the data confirmed that increasing yeast extract concentration significantly enhances GA_3_ production (*p* < 0.05).

**Figure 2:**
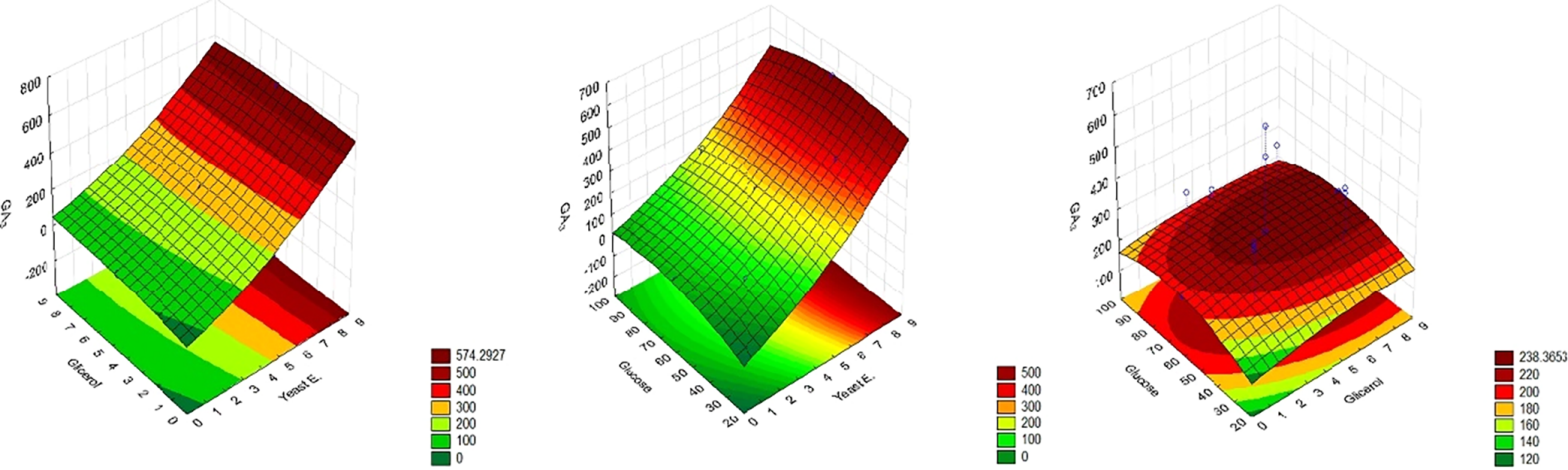
Response surface plots illustrating the effects of glucose, glycerol, and yeast extract concentrations on gibberellic acid (GA_3_) production by *Aspergillus niger*. Color gradients represent predicted GA_3_ concentrations, with red areas indicating higher production levels.

It was observed that glycerol had little influence on GA_3_ production, regardless of its concentration, if yeast extract levels were high. A similar trend was found for glucose: when combined with high concentrations of yeast extract, its effect on GA_3_ production was also minimal. When analyzing the interaction between glucose and glycerol, the region of greatest interest appeared to be at intermediate concentrations of both; however, GA_3_ production in this range remained relatively low.

Figure 3 shows the GA_3_ observed versus GA_3_ predicted plots for gibberellic acid production by *Aspergillus niger*. The points ideally form a straight line with slope 1, indicating that the model’s predictions are an unbiased match to the observations. Mean relative error (MRE) was 0.082 for both GA_3_ and residual glucose, showing the accuracy of the model used.

**Figure 3:**
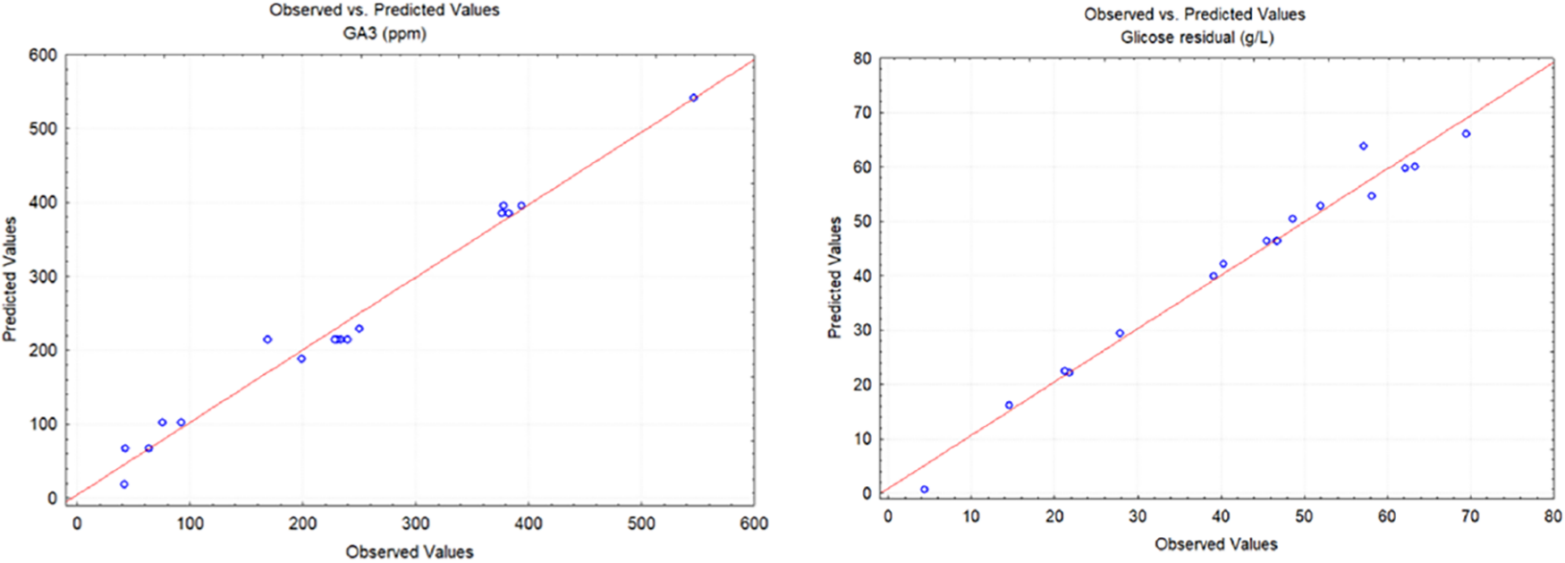
Observed vs predicted plots of the gibberellic acid production by *Aspergillus niger*.

The data obtained was subjected to desirability analysis to identify the most favorable substrate composition for maximizing GA_3_ production. As shown in Figure 4, the analysis yielded a high overall desirability score of 92%. According to the desirability analysis, the optimal formulation consisted of 26.4 g/L glucose, 4.35 g/L glycerol, and 8.7 g/L yeast extract with a predicted GA_3_ concentration of approximately 594 mg/L and minimal residual glucose. These conditions led to a notable improvement in process performance, including yield, which reached approximately 550 mg of GA_3_ per gram of glucose consumed.

**Figure 4:**
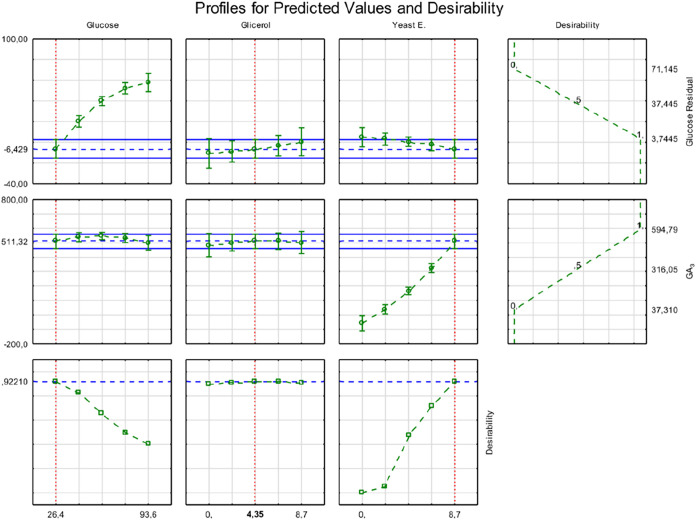
Profile plots for predicted values and desirability of gibberellic acid (GA_3_) production by *Aspergillus niger* LGB-034–2015 strain as a function of glucose, glycerol, and yeast extract concentrations. Each panel shows the predicted behavior of a response variable (residual glucose, GA_3_ production, and desirability) while varying one factor and holding the others constant. The red dashed lines indicate the optimal values for each component, and the green dotted lines represent the trends in the responses.

For the *F. fujikuroi* Insumicro 175 strain, Table 4 presents the results obtained for GA_3_, residual glucose, and the substrate yield for the CCD. The GA_3_ production values ranged from 152.29 mg/L (trial 3) to 558.45 mg/L (trial 14). The residual glucose concentrations varied between 0.00 and 46.20 g/L (trials 2 and 4) and 0.00 and 4.10 g/L (trial 7). The product yield with glucose (*Y*_P/S_) ranged from 5.10 mg/g (trial 3) to 13.67 mg/g (trial 11).

**Table 4:** Central Composite Design Results for *Fusarium fujikuroi* Insumicro 175 Strain, Evaluating the Effects of Glucose, Glycerol, and Yeast Extract Concentrations on Gibberellic Acid (GA_3_) Production, Residual Glucose, and Product Yield (*Y*_P/S_) with Glucose^a^

Trial	Glucose (g/L)	Glycerol (g/L)	Yeast extract (g/L)	GA_3_ (mg/L)	Residual glucose (g/L)	*Y*_P/S_ (mg/g)
1	40.0	2.0	2.0	208.46	2.92	6.88
2	40.0	2.0	7.0	506.43	0.00	13.5
3	40.0	7.0	2.0	152.29	1.93	5.10
4	40.0	7.0	7.0	389.11	0.00	10.6
5	80.0	2.0	2.0	243.45	37.7	5.76
6	80.0	2.0	7.0	520.74	30.1	8.62
7	80.0	7.0	2.0	183.03	46.2	5.42
8	80.0	7.0	7.0	459.91	27.0	9.59
9	26.4	4.5	4.5	229.75	0.550	8.72
10	93.6	4.5	4.5	392.32	39.2	7.21
11	60.0	0.0	4.5	469.90	29.1	13.7
12	60.0	8.7	4.5	279.97	27.2	8.54
13	60.0	4.5	0.0	191.38	40.5	8.31
14	60.0	4.5	8.7	558.45	3.08	9.81
15 (C)	60.0	4.5	4.5	307.30	8.48	6.51
16 (C)	60.0	4.5	4.5	296.18	13.8	6.41
17 (C)	60.0	4.5	4.5	305.16	19.0	6.56

a GA_3_, gibberellic acid; CP, central point; Y_P/S_, product yield with glucose.

For experiments using low initial glucose concentration (40 g/L), the GA_3_ production ranged between 152.29 and 506.43 mg/L. For high glucose concentration (80 g/L), values ranged from 183.03 to 520.74 mg/L. Intermediate glucose levels (60 g/L) combined with different concentrations of glycerol and yeast extract resulted in GA_3_ production ranging from 191.38 to 558.45 mg/L.

Trials 9 and 10 correspond to axial levels of glucose (26.4 and 93.6 g/L, respectively), resulting in GA_3_ concentrations of 229.75 and 392.32 mg/L. Trials 11 and 12 evaluated the axial limits of glycerol (0 and 8.7 g/L), while trials 13 and 14 corresponded to the minimum and maximum values of yeast extract (0 and 8.7 g/L). The central point was tested in triplicate (trials 15–17), resulting in GA_3_ concentrations of 296.18 to 307.30 mg/L, residual glucose ranging from 8.48 to 19.00 g/L, and *Y*_P/S_ values between 6.41 and 6.56 mg/g, demonstrating the reproducibility of the experimental design.

The response surface plots in Figure 5 illustrate the effects of the independent variables (glucose, glycerol, and yeast extract) on GA_3_ production by the *F. fujikuroi* Insumicro 175 strain. In the interaction between glucose and glycerol (Figure 4, top left), the GA_3_ production increased with higher glucose concentrations, especially at low to intermediate levels of glycerol. As the glycerol concentration increased, we observed that the GA_3_ production decreased across the glucose range.

**Figure 5:**
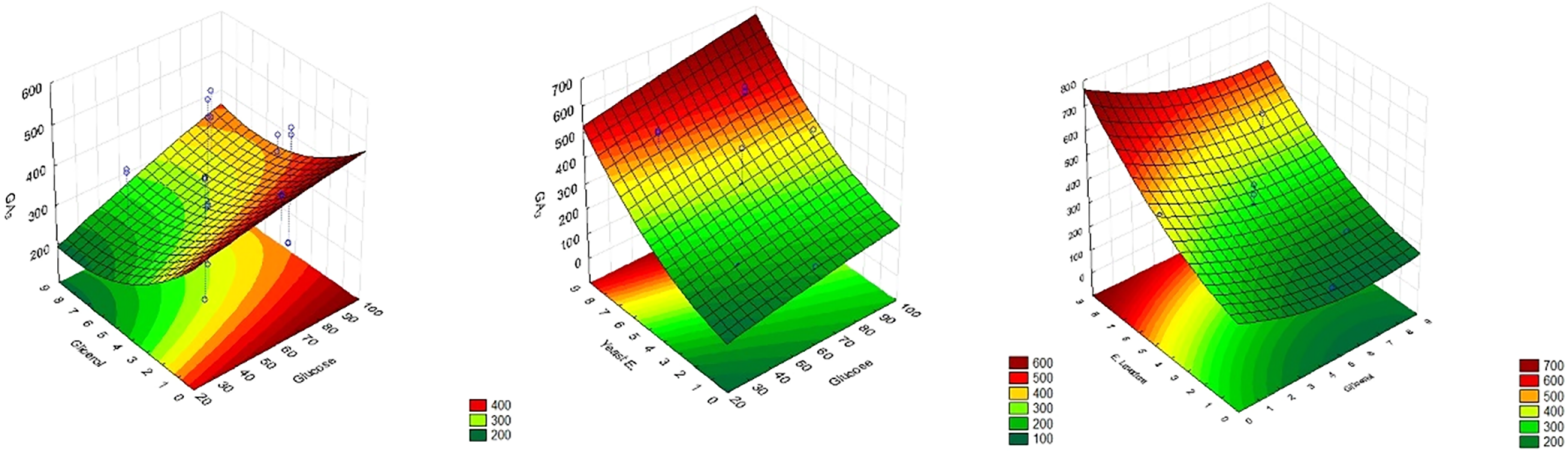
Response surface plots showing the effects of glucose, glycerol, and yeast extract concentrations on gibberellic acid (GA_3_) production by *Fusarium fujikuroi* strain Insumicro 175. (Top left) Interaction between glucose and glycerol; (top right) interaction between glucose and yeast extract; (bottom center) interaction between glycerol and yeast extract. GA_3_ concentration (mg/L) is represented by the color scale, with red indicating the highest production levels and green the lowest.

In the interaction between glucose and yeast extract (top right), a similar trend was observed: increasing yeast extract concentrations led to greater GA_3_ production, particularly at higher glucose concentrations. The highest levels of GA_3_ were found when both glucose and yeast extract were present in elevated amounts.

The interaction between glycerol and yeast extract (bottom center) showed that increasing yeast extract levels enhanced GA_3_ production, regardless of glycerol concentration. However, lower glycerol concentrations still resulted in slightly higher GA_3_ levels overall.

Figure 6 also shows the GA_3_ observed versus GA_3_ predicted plots for gibberellic acid production by *Fusarium fujikuroi*. The points ideally form a straight line with slope 1, indicating that the model’s predictions are an unbiased match to the observations. MRE was 0.06587 for the GA_3_ response, showing the accuracy of the model, too. The slope of the regression of PO values of residual glucose presented in Figure 6 differed from 1. Looking at the graph, we can state that the model overestimated observed data at low values and underestimated it at high values; thus, the slope of the regression was different from 1. The MRE between the predicted and observed values for residual glucose was 1.47.

**Figure 6:**
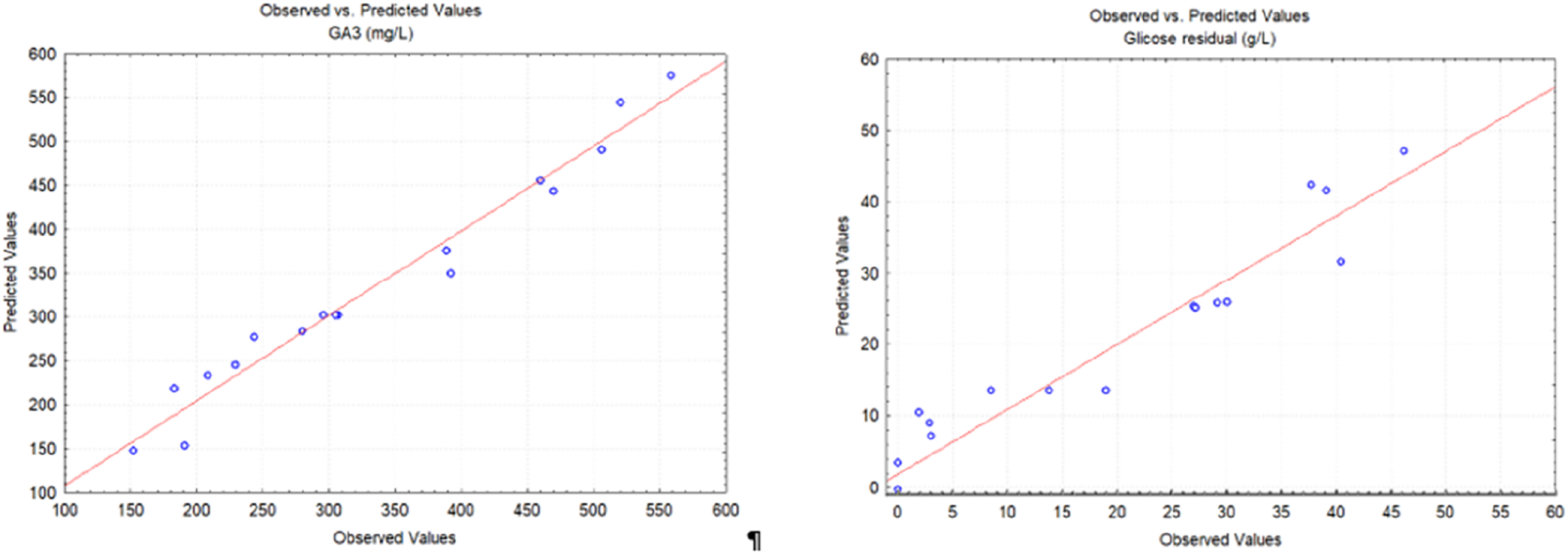
Observed vs predicted plots of the gibberellic acid production by *Fusarium fujikuroi*.

Figure 7 shows the predicted response profiles and the desirability function for GA_3_ production by the *F. fujikuroi* Insumicro 175 strain as a function of glucose, glycerol, and yeast extract concentrations. In the first row, GA_3_ production shows a slight increase with increasing glucose concentrations, a decrease with increasing glycerol, and a strong positive response to increasing yeast extract levels. The optimal predicted GA_3_ concentration was approximately 591.66 mg/L.

**Figure 7:**
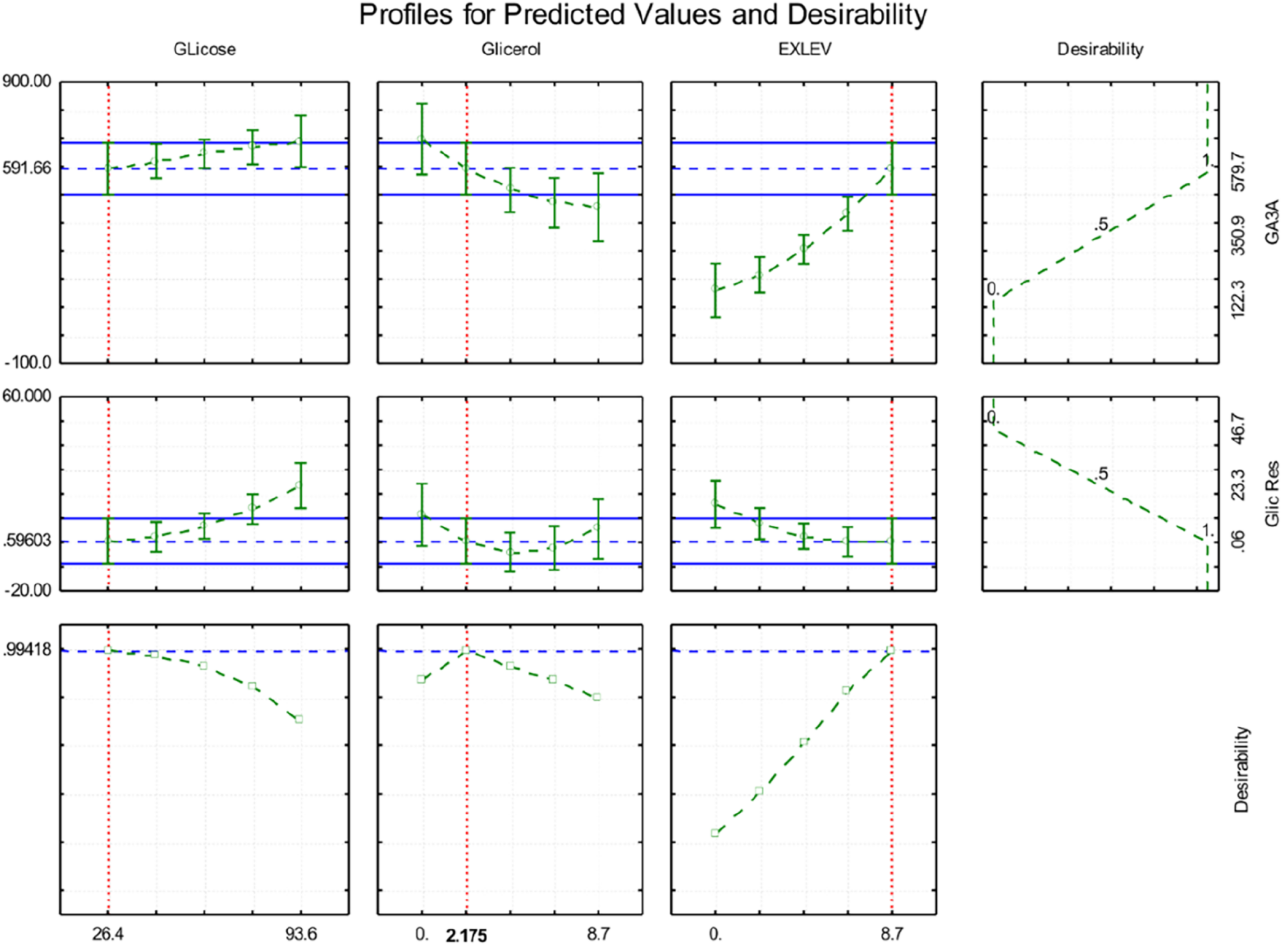
Predicted profiles and desirability analysis for gibberellic acid (GA_3_) production by *Fusarium fujikuroi* strain Insumicro 175. The plots represent the influence of glucose, glycerol, and yeast extract (EXLEV) concentrations on GA_3_ production (top row), residual glucose (middle row), and desirability function (bottom row). Dashed red lines indicate optimal values for each component. Blue dashed lines represent prediction intervals, and the green dotted lines show response trends.

The second row presents residual glucose levels. Residual glucose increases with higher glucose concentrations and remains nearly constant across the glycerol and yeast extract gradients, indicating a limited impact of these two variables on sugar consumption.

The third row illustrates the desirability function, combining the goals of maximizing GA_3_ production with minimizing residual glucose. Desirability was maximized at a value of 0.994 with the combination of 26.4 g/L glucose, 2.17 g/L glycerol, and 8.700 g/L yeast extract. Under these conditions, the predicted GA_3_ production was approximately 580 mg/L with a low residual glucose concentration.

### 3.2 Validation Assays of the Substrate Designed by Desirability Analysis

Validation assays were carried out in triplicate for both fungal strains using the optimized substrate compositions predicted by the desirability analysis. The experiments followed the same methodology previously described. For *A. niger*, the achieved GA_3_ medium concentration was 5.97 × 10^2^ ± 4.71 × 10^1^ mg/L, closely matching the model-estimated value of 5.95 × 10^2^ mg/L, with a relative deviation of 3.75 × 10^–1^%.

In contrast, *F. fujikuroi* achieved a GA_3_ medium concentration of 4.87 × 10^2^ ± 1.11 × 10^1^ mg/L, compared to a model-estimated value of 5.79 × 10^2^ mg/L. The relative deviation was 1.59 × 10^1^%.

### 3.3 Effect of the Produced Supernatant on Plant Stress Physiology and Bean Yield

After evaluation under controlled conditions, the agronomic effect of the produced supernatant was assessed under field conditions to validate the positive responses previously observed. Table 5 summarizes the results obtained from the common bean crop after foliar application of the supernatant at different doses.

**Table 5:** Results Obtained for Lipid Peroxidation, Total Soluble Protein, Enzymatic Activity, Thousand-Grain Weight, and Productivity^a^,^b^

Treatment	LP	TSP	SOD	POD	CAT	TGW (g)	Productivity (bags/ha)
Control	15.70^b^	5.85^a^	85.830^c^	3.78^b^	243.53^b^	203.58^a^	39.56^b^
Positive control	19.98^a^	4.66^b^	109.58^b^	4.20^b^	277.33^b^	200.18^a^	34.20^b^
SF 250	15.75^b^	2.90^c^	238.05^a^	7.10^a^	437.88^a^	198.43^a^	37.00^b^
SF 500	12.03^c^	6.73^a^	91.750^c^	3.00^c^	229.93^b^	197.58^a^	37.93^b^
SF 1000	11.10^c^	5.88^a^	106.85^b^	2.78^c^	103.63^c^	192.88^a^	37.70^b^
SF 2000	11.25^c^	5.85^a^	105.98^b^	2.75^c^	145.90^c^	209.18^a^	44.83^a^
% CV	7.73	15.02	11.010	18.10	18.840	4.160	8.820

a Productivity is expressed in bags per hectare (bags/ha, 60 kg bags).

b LP, lipid peroxidation; TSP, total soluble protein; SOD, superoxide dismutase; POD, peroxidase; CAT, catalase; TGW, thousand-grain weight; CV, coefficient of variation. Means followed by the same lowercase letter do not differ statistically by the Scott–Knott test (*p* < 0.05). Means followed by the same letter do not differ significantly according to the Scott–Knott test at the 5% significance level. The letters a–c represent statistically distinct.

LP (nmol TBARS gMF^–1^) showed statistically significant differences (*p* < 0.05) among treatments. The positive control presented the highest value, followed by the control treatment. The treatment with 250 mL/ha of the supernatant showed values statistically similar to the control. All higher doses of the supernatant (500, 1000, and 2000 mL/ha) resulted in significantly lower lipid peroxidation values.

TSP varied among treatments, with the lowest value observed in the 250 mL/ha treatment, which differed significantly from those of the others. All other treatments showed values statistically similar to those of the control.

Regarding antioxidant enzyme activity, significant differences (*p* < 0.05) were observed for SOD, POD, and CAT. The 250 mL/ha treatment showed the highest enzymatic activity for all three enzymes. Treatments with 500, 1000, and 2000 mL/ha presented intermediate or lower values depending on the enzyme analyzed.

TGW did not differ significantly among treatments; all of the means were statistically similar. Productivity (bags/ha, 60 kg bags) differed only for the treatment with 2000 mL/ha of the supernatant, which showed a significantly higher mean compared to the other treatments.

Table 6 shows the current GA_3_ results compared with the other published results.

**Table 6:** Summary of GA_3_ Production Results for Different Microorganisms and Fermentation Conditions for Comparison with the Research Results

Microorganism (strain)	Fermentation method	Main substrate	Maximum GA_3_ production	Source (and manuscript citation)
*Aspergillus niger* LGB-034–2015	Submerged	Glucose, glycerol, and yeast extract (optimized)	597.03 mg/L	Present study
*Fusarium fujikuroi* Insumicro 175	Submerged	Glucose, glycerol, and yeast extract (optimized)	487.23 mg/L	Present study
*Fusarium moniliforme*	Submerged	Banana peel waste	17.480 mg/L	Omojasola and Adejoro^21^
*Aspergillus niger*	Submerged	Banana peel waste	13.500 mg/L	Omojasola and Adejoro^21^
*Fusarium* sp.	Submerged	Glycerol-enriched medium	2.800 mg/L	Peng et al.^17^
*Aspergillus niger*	Solid-state	Corn cob residues	≈6.100 mg/L	Monrroy and García^11^
*Aspergillus flavus* Y2H001	Liquid	Standard culture medium	1.954 × 10^–3^ mg/L	Rhu et al.^18^

## 4 Discussion

In this study, we demonstrated that the fermentation supernatant produced by *Aspergillus niger* LGB-034–2015 and *Fusarium fujikuroi* Insumicro 175 has potential for biotechnological use, increasing GA_3_ production and improving physiological responses in several crops. We evaluated GA_3_ production and yield in strains of *A. niger* and *F. fujikuroi* to identify new biological sources and to expand opportunities for agronomic applications.

The substantial increase in GA_3_ production and yield observed in several trials highlights the positive impact of combining the studied factors at specific concentrations, with some conditions resulting in approximately 550 mg/L GA_3_ per gram of glucose consumed. This high conversion efficiency reinforces the importance of medium optimization for increasing total metabolite concentration and for enhancing the process yield, a key parameter for industrial applications.

Previous studies have also attempted to optimize GA_3_ production under similar conditions, but with significantly different outcomes.^22–26^ In the study reported by Rhu and colleagues,^18^ *A. flavus* produced GA_3_ at concentrations in the nanogram per milliliter range 1.954 ng/mL (1.954 × 10^–3^ mg/L), a markedly lower scale when compared to the values obtained in the present study with *A. niger*. This vast difference in production levels can be attributed to multiple factors, including the specific *Aspergillus* species and strains, the extent and design of the optimization process, and the nutritional composition of the fermentation medium.

Thus, the results from *A. niger* LGB-034–2015 in this study demonstrate the effectiveness of the optimization strategy, as well as the biotechnological potential of the selected strain for high-yield GA_3_ production.

Regarding the *F. fujikuroi* Insumicro 175 fermentation, we reached a yield of approximately 550 mg/L GA_3_ per gram of glucose consumed. The GA_3_ concentration obtained in this study is considerably lower compared with studies that employ more intensive optimization strategies or alternative substrates.^27–30^ For instance, Peng et al.^17^ reported GA_3_ concentrations of 2.8 g/L (2.8 × 1000 mg/L) using mutant strains of *F. fujikuroi* cultivated in enriched media, although this approach involved higher production costs. These disparities underscore the considerable influence of fermentation conditions, substrate composition, and advanced bioprocess optimization techniques on achieving superior GA_3_ yields from *F. fujikuroi*.^31^ The values of GA_3_ per gram of glucose consumed in this study demonstrate the effectiveness of the optimized process, particularly considering the use of simple, low-cost substrates.

The effects of the independent variables (glucose, glycerol, and yeast extract) on GA_3_ production were distinct for the two fungal strains studied. In both *A. niger* and *F. fujikuroi*, glucose had a significant positive effect on GA_3_ production up to a certain concentration, beyond which the yield (mg of GA_3_/g of glucose) tended to plateau or slightly decrease, likely due to substrate inhibition or accumulation of residual glucose. This behavior is consistent with previous reports.^7,28,32–34^

The study indicates that the use or increase of the glycerol concentration led to a notable reduction in GA_3_ production for both strains. This finding is consistent with preliminary experiments and with the results reported by Omojasola and Adejoro,^21^ who also observed the negative impact of glycerol on GA_3_ biosynthesis. Interestingly, low concentrations of glycerol combined with yeast extract showed a synergistic effect in *A. niger*, enhancing GA_3_ levels, which was not observed in *F. fujikuroi*.

The response surface plots further support these findings by showing that at high concentrations of yeast extract, the effects of both glucose and glycerol become less pronounced. This suggests that nitrogen availability plays a dominant role in stimulating GA_3_ synthesis under the tested conditions, according to other studies that also highlighted the key role of nitrogen sources in maximizing gibberellin yields.^6,30,31^ Overall, the results highlight distinct metabolic responses between the strains, with *A. niger* exhibiting greater substrate conversion efficiency, while *F. fujikuroi* achieved comparable GA_3_ concentrations under optimized conditions, although with a lower yield per gram of glucose consumed. In this way, our results reinforce species-specific responses to substrate combinations and metabolic flexibility in *A. niger*.

Glucose also positively influenced production, although high concentrations led to an accumulation of residual sugar and a decrease in yield per gram of substrate, suggesting that carbon excess could inhibit metabolic balance.^35–37^ These findings reinforce the need for precise substrate formulation to optimize both productivity and efficiency.

The optimization performed through desirability analysis confirmed these trends and provided substrate compositions that led to high GA_3_ concentrations with low residual glucose, validating the model’s predictive power. For *A. niger*, the model suggested a yield of 595.0 mg/L GA_3_, closely matched by the experimental value of 597.03 mg/L. For *F. fujikuroi*, the predicted value was 579.00 mg/L, with the experiment reaching 487.23 mg/L. The high desirability index (92–99%) reinforces the robustness of the optimization strategy, aligning with methods reported in recent studies.^27,38,39^

In the agronomic assays, while this number of replications is standard for initial field trials, we acknowledge that a relatively low replication may be a constraint on the statistical robustness of the conclusions. Foliar application of the fermentation supernatant revealed promising biostimulant activity in common beans. Lipid peroxidation levels significantly decreased with increasing supernatant doses (up to 30%), indicating reduced oxidative stress. These results are corroborated by previous research, which observed reductions in oxidative stress response with the application of hormonal biostimulants.^40,41^ The lowest stress indices were observed with the 1000 and 2000 mL/ha treatments, demonstrating the capacity of the supernatant to mitigate reactive oxygen species (ROS) accumulation.

The enzymatic responses also reflected the plant’s adaptation to the biostimulant. SOD activity increased in nearly all treatments except SF 500. POD and CAT activities were significantly enhanced only at the lowest dose (SF 250), suggesting that at higher doses, the plant’s oxidative balance was achieved with reduced need for enzymatic activation. This behavior aligns with that reported by other authors, who found that exogenous hormone application led to lower oxidative enzyme activity due to the activation of nonenzymatic defense pathways.^42–44^

Finally, the agronomic impact of the supernatant was confirmed by a significant increase in productivity at the highest dose (2000 mL/ha), which yielded 44.8 bags/ha. Importantly, this result was statistically greater than those of both the control and the tested commercial hormone-based product. While no statistical difference was found for TGW, the highest absolute values were also recorded at this dose, suggesting improved nutrient translocation. These findings indicate the promising potential of the supernatant as a source of GA_3_ and as a multifunctional biostimulant for field application, but require broader validation against commercial standards.

The superior agronomic performance achieved with the optimized fermentation supernatant directly demonstrates the viability of the production process, particularly concerning the use of higher-cost inputs, such as yeast extract. To quantify this viability, a technological economic assessment (TEA) was conducted. The GA_3_-containing fermented product (≈600 ppm GA_3_) was applied at a rate of 2 L/ha. Based on the optimized medium composition (26.4 g/L glucose, 4.35 g/L glycerol, and 8.7 g/L yeast extract) and bulk procurement prices in Brazil, the raw material cost was estimated to be US$0.17/L (glucose: R$250/25 kg; yeast extract: R$1,700/25 kg; glycerol: R$100/5 L; density ≈1.26 kg/L) converted at 1 USD = 5.45 BRL on 16 Oct 2025. Incorporating typical industrial operating costs for aerobic fermentation and formulation (utilities/steam–electricity, labor and QA/QC, maintenance, depreciation/overhead), estimated at US$0.20/L based on early stage bioprocess TEA benchmarks,^45–47^ yields a total manufacturing cost of approximately US$0.37/L, equivalent to US$0.74/ha at the field application rate. In field trials, the product increased common bean yield by 5.27 sacks (60 kg each) ha^–1^ relative to that of the untreated control. Using the Center for Advanced Studies on Applied Economics—CEPEA reference prices for Carioca beans (screen size 12; Itapeva, R$262.11 per sack, as of October 15, 2025), the resulting gross additional revenue was approximately US$253 ha^–1^. This corresponds to a benefit–cost ratio of ∼343 and a net return on investment exceeding 34,000% relative to manufacturing costs. The break-even yield response under these conditions was minimal (∼0.015 sack ha^–1^, or ∼0.92 kg ha^–1^), indicating substantial robustness to market variability. These results conclusively demonstrate that the optimization strategy, although employing a relatively high-cost nitrogen source to maximize yield, leads to an exceptionally cost-effective and commercially viable process.

Despite the promising results, some limitations should be acknowledged. Although the strains showed high GA_3_ concentrations under optimized conditions, their stability across successive subcultures was not evaluated. Moreover, the GA_3_ purity in the dried supernatant was not determined; therefore, contributions from other fungal metabolites, such as polysaccharides, peptides, and amino acids, cannot be excluded. Field validation was conducted in a single season and location; broader validation is needed. The paradoxical response between enzyme activity and yield at different doses further indicates that complex hormonal interactions may be involved, although in-plant GA_3_ quantification and ROS imaging were not performed. In addition, the tested doses were broad and did not allow an estimated dose to specify an effect in 50% of the population, ED50, or fine-tuned dose–response curves. Finally, the observed efficacy at relatively low GA_3_ doses compared with commercial formulations suggests that other bioactive metabolites may also play a role, warranting future metabolomic profiling. These limitations highlight opportunities for deeper investigations to strengthen the robustness, reproducibility, and applicability of this approach under diverse agricultural conditions.

## 5 Conclusions

The present study demonstrated the potential of *A. niger* and *F. fujikuroi* strains for the efficient production of GA_3_ using low-cost substrates under submerged fermentation. Through response surface methodology and desirability analysis, optimized conditions were identified that significantly enhanced the GA_3_ yield, particularly in *A. niger*, which exhibited superior substrate conversion efficiency. The fermentation supernatants showed promising GA_3_ concentrations and exhibited biostimulant activity in field trials with common beans, reducing oxidative stress markers and increasing productivity. Moreover, our results demonstrated substantial agronomic benefits under field conditions. This dual functionality positions the process as a viable alternative to conventional GA_3_ production methods and a promising, sustainable source of bioactive compounds for agricultural applications. Future studies should also explore the metabolomic profile of the supernatant and assess its application in different crop systems.

## Data Availability

The data will not be shared because all the data was in the manuscript.
